# One-Pot Synthesis of Enzyme and Antibody/CaHPO_4_ Nanoflowers for Magnetic Chemiluminescence Immunoassay of *Salmonella enteritidis*

**DOI:** 10.3390/s23052779

**Published:** 2023-03-03

**Authors:** Xingchu Mao, Ranfeng Ye

**Affiliations:** College of Science, Huazhong Agricultural University, Wuhan 430070, China

**Keywords:** bioinspired, hybrid nanoflowers, magnetic chemiluminescence immunoassay, *Salmonella enteritidis*, detection, biosensing

## Abstract

In this study, through a bioinspired strategy, the horseradish peroxidase (HRP) and antibody (Ab) were co-embedded into CaHPO_4_ to prepare HRP-Ab-CaHPO_4_ (HAC) bifunctional hybrid nanoflowers by one-pot mild coprecipitation. The as-prepared HAC hybrid nanoflowers then were utilized as the signal tag in a magnetic chemiluminescence immunoassay for application in the detection of *Salmonella enteritidis* (*S. enteritidis*). The proposed method exhibited excellent detection performance in the linear range of 10–10^5^ CFU/mL, with the limit of detection (LOD) of 10 CFU/mL. This study indicates great potential in the sensitive detection of foodborne pathogenic bacteria in milk with this new magnetic chemiluminescence biosensing platform.

## 1. Introduction

With the continuous improvement of people’s living standards, food safety issues have been widely discussed and emphasized by the public. A significant percentage of foodborne diseases and deaths are ascribed to bacterial pathogens, according to the World Health Organization’s report [1,2]. *Salmonella* is well known to be the one of the most prevalent foodborne pathogenic bacteria, which can cause food poisoning and acute gastroenteritis or other health problems [3]. In order to opportunely and effectively monitor the possible outbreak of an infection in the future, it is very important to develop a reliable technology for the rapid and sensitive detection of *Salmonella*.

Currently, there are two main types of methods for the detection of *Salmonella*. One is traditional cultivation, and the other is the rapid detection methods. The traditional cultivation method has the merits of uncomplicated equipment and good reliability, but suffers from the shortcoming of time-consuming preparation procedures, including enrichment and colony isolation [4]. For the rapid detection methods, such as matrix-assisted laser desorption ionization time-of-flight mass spectrometry [5] and colony-scattering-based optical methods [6,7], these techniques are more suitable for the enumeration or identification of *Salmonella* and still have complexity in sample preparation. Despite the relatively rapid quantification methods based on immunological assays and nucleic acid probes, such as enzyme-linked immunosorbent assay (ELISA) and polymerase chain reaction (PCR), they still face the challenge of improving their detection sensitivity without enrichment [8,9,10].

A biosensor is an analytical device that is commonly used to determine the concentrations of substances or specific parameters of biological interest. With simple platforms such as lateral flow [11] and smartphones [12], etc., the biosensor has great potential for the rapid detection of *Salmonella*, allowing its use by nonscientific/nontechnical personnel and detection in different food samples (meat, milk, eggs, vegetables, etc.). For non-antibody- or non-enzyme-based biosensors, such as aptamer-based biosensors [13] and surface positively charged nitrogen-rich carbon nanoparticle-based biosensors [14], they offer acceptable affinity and sensitivity to the targets. Nanotechnology plays a very important role in the development of biosensors. The specificity and sensitivity of a biosensor can be significantly improved by employing antibody-(Ab) or enzyme-functionalized nanomaterials for its construction [15,16,17]. The functionalization of nanomaterials by conjugating Ab or enzyme proteins is usually achieved by covalent attachment or physical adsorption on presynthesized nanomaterials, which is the key to realizing efficient recognition and signal amplification [18,19]. However, these two routes for conjugation have the disadvantages of easy detachment, a loss in enzyme activity or stability or cumbersome operation and purification processes [20].

Recently, a newly developed bioinspired strategy for the synthesis of protein–inorganic hybrid nanoflowers has become an efficient solution for the conjugation of nanomaterials with proteins. Working through this methodology, various enzymes, such as amylase [21], laccase [22], sucrase [23], glucose oxidase [24], horseradish peroxidase (HRP) [25] and protease [26], etc., can be embedded with metal phosphates (e.g., Cu_3_(PO_4_)_2_) or metal hydrogen phosphates (e.g., CaHPO_4_) by one-pot mild coprecipitation to form diversified hybrid nanoflowers with simplified operation and improved enzyme activity or stability. In addition, these protein–inorganic hybrid nanoflowers can simultaneously conjugate two different proteins, such as Ab and enzymes, and have been successfully applied in the sensing of various analytes.

At present, there are many techniques combined with immunoassays, including the surface-enhanced Raman scattering [27,28,29], surface plasmon resonance [30,31], fluorescence [32,33], chemiluminescence [34], electrochemistry [35], colorimetry [36], magnetic beads [37] and phage-based technologies [38], which have been developed for the biosensing of pathogens owing to their advantages of simplicity, sensitivity, specificity or affording readout signals. In particular, chemiluminescence immunoassays have higher sensitivity, a wider dynamic range and a faster reaction rate than other traditional analytical methods [39]. However, to the best of our knowledge, the combination of such hybrid nanoflowers and magnetic chemiluminescence immunoassays for the detection of *S. enteritidis* has not yet been developed. Therefore, in this study, the HRP and Ab were co-embedded into CaHPO_4_ to prepare HRP-Ab-CaHPO_4_ (HAC) bifunctional hybrid nanoflowers by one-pot mild coprecipitation. The morphology, size, composition and structure of the prepared HAC hybrid nanoflowers were carefully characterized. Under optimized conditions, the as-prepared HAC hybrid nanoflowers then were utilized as the signal tag in a magnetic chemiluminescence immunoassay for application in the detection of S. enteritidis. The performance, including the sensitivity, specificity, accuracy and applicability, of the proposed method was also studied and discussed in detail.

## 2. Materials and Methods

### 2.1. Reagents and Materials

Calcium chloride (CaCl_2_), dimethylformamide (DMF), sodium dihydrogen phosphate (NaH_2_PO_4_), fluorescein isothiocyanate isomer I (FITC), sodium monohydrogen phosphate (Na_2_HPO_4_), dimethylsulfoxide (DMSO), HRP, luminol, 4-iodophenol and streptavidin-coated magnetic beads (SMB, diameter 2.8 μm) were purchased from Sigma-Aldrich. Polycolonal antibody (pAb) and biotinylated monoclonal antibody (mAb) to *S. enteritidis* were obtained from the KPL company in the United States. *S. enteritidis* 50041, *Listeria monocytogenes* 54004 and *E. coli* O157:H7 44939 were from the Chinese National Center for Medical Culture Collections (CMCC). Sulfo-cyanine5 (Cy5) *N*-hydroxysuccinimide ester was purchased from the Lumiprobe company in the United States.

### 2.2. Synthesis and Characterization of HAC

The synthesis of HAC was conducted according to a previous study, with modification [40]. In a typical synthesis procedure, 980 μL phosphate-buffered saline (PBS) (3 mM, pH 6.8) containing 181.8 μg HRP and 18.18 μg pAb was mixed with 20 μL 200 mM CaCl_2_ solution to react at room temperature for 12 h. The obtained product was centrifuged at 10,000× *g* rpm for 5 min, and then washed with deionized water 3 times. The purified product was dispersed in PBS. A drop of the obtained product was dried naturally on a filter membrane and then treated by gold spraying for the scanning electron microscope (SEM, JSM-6390LV, NTC Ltd., Tokyo, Japan) data collection. A drop of the obtained product was dried naturally on a carbon grid for the transmission electron microscope (TEM, Hitachi H-7650, Hitachi High-Technologies Co., Tokyo, Japan) data collection. The obtained product was treated by vacuum freeze-drying for the X-ray diffraction (XRD, Rigaku MiniFlex 600 Rigaku Co., Tokyo, Japan) analysis. A drop of the obtained product was dried naturally on a glass slide for fluorescence confocal microscopy (Leica TCS SP5, Leica Microsystems GmbH, Wetzlar, Germany) data collection.

### 2.3. Synthesis of HRP-Cy5 and pAb-FITC Conjugate

The synthesis was conducted according to a previous study, with modification [41]. The HRP-Cy5 conjugate was synthesized as follows: 100 μL DMF containing Sulfo-Cyanine5 NHS ester (0.30 mg) was mixed with 900 μL 0.1 M sodium bicarbonate solution containing 10 mg HRP and allowed to react in the fridge at 4 °C overnight. The obtained HRP-Cy5 conjugate was purified by centrifugal devices (Nanosep) with ultrafiltration membranes (3000 molecular weight cut-off). The pAb-FITC conjugate was synthesized as follows: 5 μL DMSO containing 1 mg/mL FITC was mixed with 100 μL 2 mg/mL pAb to react in the fridge at 4 °C for 12 h. The obtained pAb-FITC conjugate was purified by centrifugal devices (Nanosep) with ultrafiltration membranes (3000 molecular weight cut-off).

### 2.4. Immobilization Efficiency of HRP and pAb in HAC

The procedure was followed according to a previous study, with modification [41]. In order to calculate the immobilization efficiency of HRP and pAb in HAC hybrid nanoflowers, a series of 3 mM PBS containing pAb-FITC with concentrations of 0, 1, 10, 15 and 25 μg/mL were prepared to establish a standard curve of correlation between the concentration of pAb-FITC and the fluorescence intensity. The fluorescence intensity of pAb-FITC was detected at 525 nm. A series of 3 mM PBS containing HRP-Cy5 with concentrations of 0, 50, 100, 150 and 250 μg/mL were prepared to establish a standard curve of correlation between the concentration of HRP-Cy5 and the fluorescence intensity. The fluorescence intensity of HRP-Cy5 was detected at 670 nm. The amount of HRP immobilized on HAC was determined in the following way: the fluorescence intensity of free HRP-Cy5 in the supernatant (10 μL) of the as-prepared HAC solution before washing with deionized water was measured to determine the amount of free HRP-Cy5 based on the standard curve. The amount of immobilized HRP was the difference value between the total amount of HRP-Cy5 added and the amount of free HRP. The immobilization efficiency of pAb-FITC was determined in the same way.

### 2.5. Preparation of SMB-Conjugated mAb (SMB-mAb)

The SMB-mAb was used as a capture probe and prepared as follows: 400 μL 5 mg/mL SMB was mixed with 100 μL 1 mg/mL biotinylated mAb prepared with PBS and incubated for 30 min in a rotary mixer at room temperature. The resulting SMB-mAb was washed with PBS containing 0.1% BSA four times and reconstituted with 500 μL PBS and stored in the fridge at 4 °C.

### 2.6. Procedure for S. enteritidis Detection

For the biorecognition of the target of *S. enteritidis*, 5 μL SMB-mAb was mixed with 10 μL of different concentrations of *S. enteritidis*. The mixture was incubated for 60 min at 35 °C and magnetically separated (by magnetic separation rack with 96 wells, Beyotime Biotech. Inc., Shanghai, China) by washing with PBS three times. The supernatant was totally removed and 50 μL synthesized HAC with an appropriate dilution (dilution buffer: PBS containing 1% bovine serum protein (BSA) and 0.5% Tween-20) was added before incubation for 30 min at 35 °C. The unbound HAC was removed by magnetic separation and washed with PBS three times. Then, 100 μL of a chemiluminescent substrate solution (2 mM luminol, 4 mM H_2_O_2_ and 0.5 mM *p*-iodophenol dissolved in 0.1 M pH 8.5 Tris–HCl) was added and mixed in the microplate to catalyze the chemiluminescent reaction. The chemiluminescent signal was measured using the SpectraMax i3 microplate reader (Molecular Devices, Sunnyvale, CA, USA). The relative chemiluminescent intensity (RCI) was represented as C/C_0_, where C and C_0_ are the RCI in the presence and absence of *S. enteritidis*, respectively.

### 2.7. Selectivity and Recovery Experiment

Two non-target bacteria of *E. coli* O157:H7 and *Listeria monocytogenes* and the mixture of *S. enteritidis* and two non-target bacteria were determined to investigate the specificity of this method (all bacteria at the same concentration of 1 × 10^4^ CFU/mL). For recovery experiments, milk purchased from a local supermarket was spiked with *S. enteritidis* at the concentrations of 1 × 10^2^, 1 × 10^3^ and 1 × 10^4^ CFU/mL, which were detected based on the standard curve.

## 3. Results and Discussion

### 3.1. Preparation and Sensing Principle of HAC Nanoflowers

**Scheme 1:**
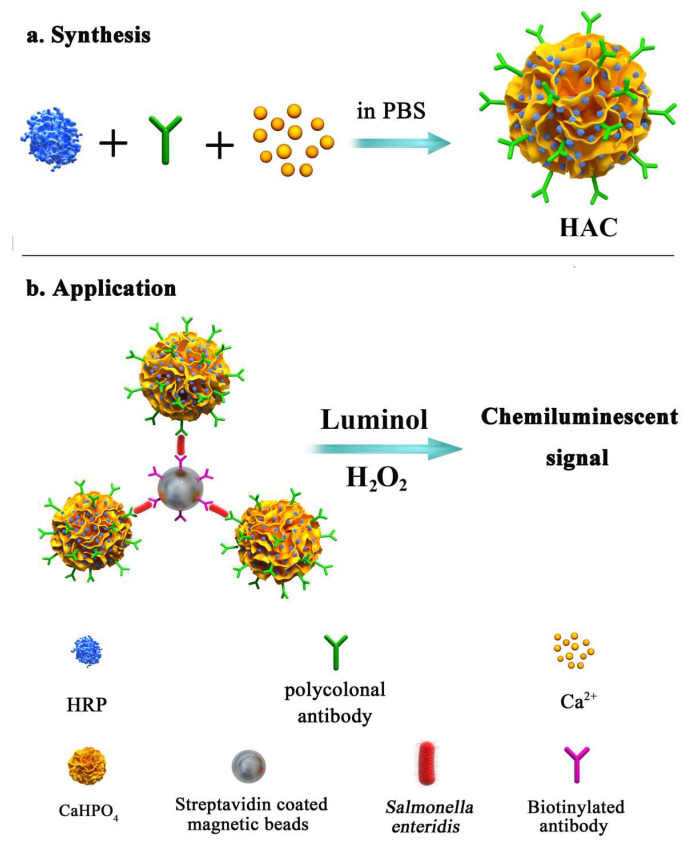
Illustration of the preparation process for the HAC nanoflowers and their application in the detection of *S. enteritidis*. (**a**) The synthsis of HAC; (**b**)The application in the detection of *S. enteritidis*.

Scheme 1 depicts the mechanism and process of the synthesis of HAC nanoflowers and their application in the magnetic chemiluminescence immunoassay of *S. enteritidis*. The formation of HAC organic–inorganic hybrid nanoflowers was similar to the biomineralization. The Ca^2+^ could react with HPO_4_^2−^ to form CaHPO_4_ in PBS. Due to the introduction of organic components (HRP and pAb) in the reaction system, the Ca^2+^ could also form a complex with protein (HRP and pAb) molecules via their carboxylic groups [41], and then served as the nucleation site for the formation of primary nanoparticles of CaHPO_4_ that were employed as the inorganic component and provided a biocompatible interface for the efficient immobilization of HRP and pAb by one-pot coprecipitation. The synthesized HAC nanoflowers were then applied as the signal tags in the magnetic chemiluminescence immunoassay for the detection of *S. enteritidis*. A large amount of immobilized HRP on HAC executed the function of signal amplification and the pAb on HAC ensured the recognition of the target *S. enteritidis*. After the formation of a sandwich immunocomplex and magnetic separation, the indirect chemiluminescence signal was produced based on the oxidation of luminol by hydrogen peroxide catalyzed by HRP, with *p*-iodophenol as an enhancer, which exhibited maximal emission around 425 nm [42]. The intensity was proportional to the concentration of *S. enteritidis*, which allowed the establishment of a standard curve for the quantitative detection of *S. enteritidis*.

### 3.2. Characterization of HAC Nanoflowers

**Figure 1:**
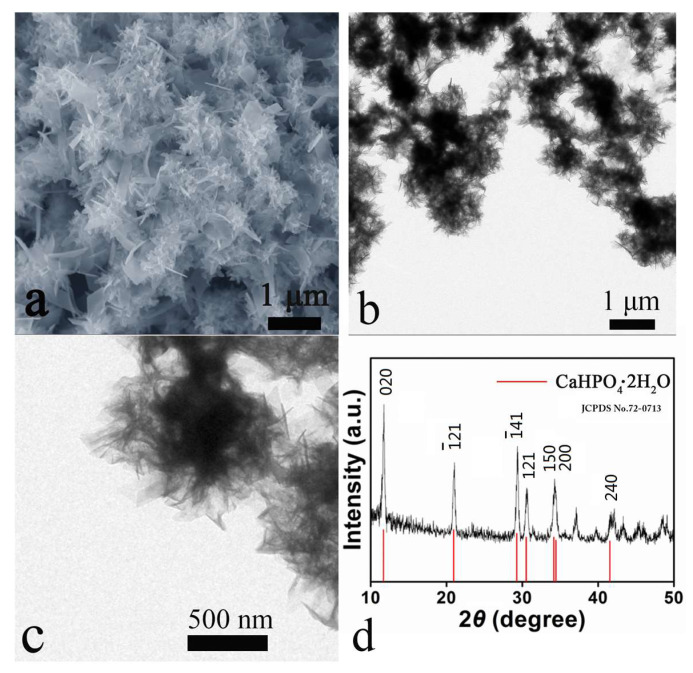
(**a**) SEM image of HAC nanoflowers; (**b**) TEM image of HAC nanoflowers; (**c**) TEM image of HAC nanoflowers (partial magnification from (**b**)); (**d**) XRD pattern of HAC nanoflowers (JCPDS No. 72–0713).

**Figure 2:**
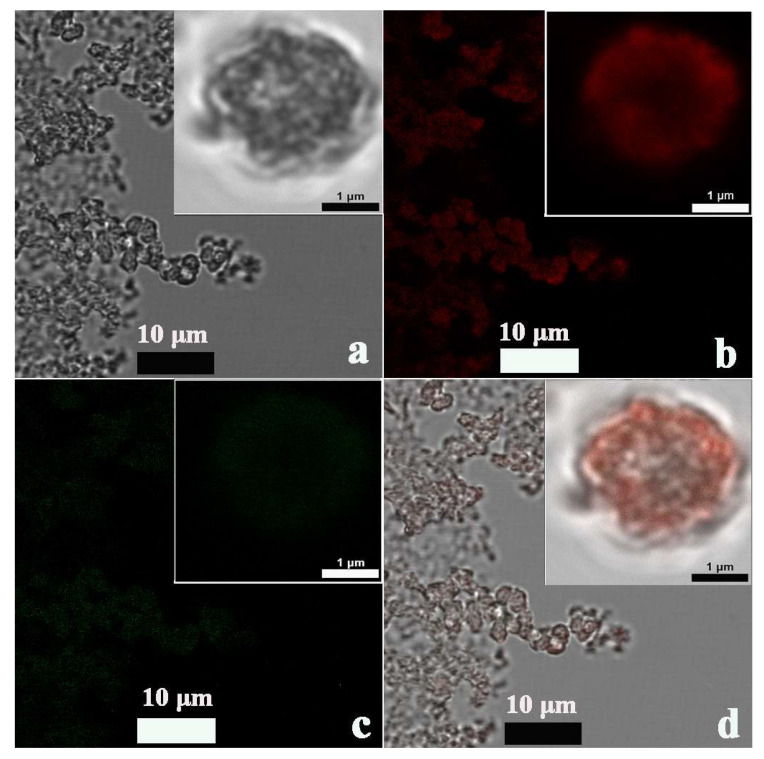
Confocal fluorescence microscopy images of HAC. (**a**) Bright-field image of HAC; (**b**) Cy5-labeled HRP in HAC; (**c**) FITC-labeled pAb in HAC; (**d**) overlap image of bright-field and fluorescence images. Insert is the magnification of a single HAC.

To investigate their size and morphology, the HAC were firstly characterized by SEM and TEM (Figure 1). The SEM image showed that the shape of the HAC nanohybrids was flower-like, with a size of ~1.5 um (Figure 1a). The same observation was also verified by the TEM images, and the petal-like structure in the HAC nanoflowers seemed to be composed of nanosheets (Figure 1b,c). XRD analysis was performed to study the crystalline structure and composition of the HAC nanoflowers. The XRD patterns had good agreement with the characteristic peaks of CaHPO_4_·2H_2_O (Figure 1d), which confirmed the inorganic composition of the HAC nanoflowers. In addition to the elements of calcium, oxygen and phosphorus detected by the dispersive X-ray (EDX) analysis (Figure S1, Supporting Materials), the element of carbon was also found, indicating that the organic component of HRP and pAb could be hybridized with the CaHPO_4_. To corroborate this, HRP and pAb were both immobilized in the HAC nanoflowers, and the HRP and pAb were fluorescently labeled with Cy5 and FITC, respectively, to monitor the synthesis of HAC (Figure 2). The characteristic fluorescence of Cy5 (red, Figure 2b) and FITC (green, Figure 2c) was displayed in the fluorescence confocal microscopy images, suggesting the successful coimmobilization of HRP and pAb in the HAC nanoflowers. The immobilization efficiency of HRP and pAb in HAC was determined to be 25.1% and 65.7%, respectively, based on an established standard curve of correlation between the concentration of HRP-Cy5 (or pAb-FITC) and the fluorescence intensity.

### 3.3. Optimization of Detection Conditions

**Figure 3:**
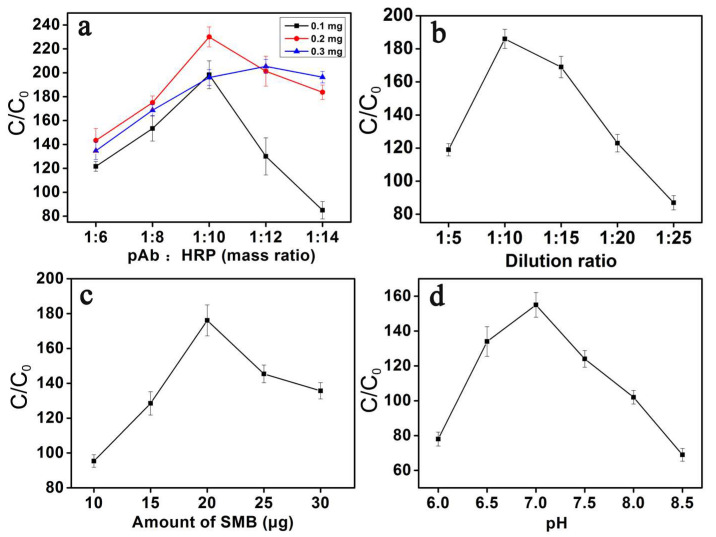
Optimization of detection conditions. (**a**) Different total amounts of HRP and pAb and their mass ratios for HAC preparation. (**b**) Different dilution ratios of HAC for detection. (**c**) Different amounts of SMB for detection. (**d**) Different pH values for HAC binding.

To acquire improved analytical performance, several parameters of the experiment were optimized (Figure 3). Firstly, the amount of HRP immobilized on the HAC nanoflowers was directly related to the catalytic efficiency and RCI. The amount of pAb affected the recognition efficiency for the target *S. enteritidis* and was indirectly related to the RCI. Thus, the total amount of HRP and pAb and their mass ratio played an important role in the sensitivity of this method. As shown in Figure 3a, a significant increase in RCI occurred within the mass ratio of pAb to HRP from 1:6 to 1:10, and it then reached the strongest RCI at 1:10 with the total amount of 0.2 mg. Other proportions and total amounts of pAb and HRP could not achieve a higher RCI, which may due to the lack of sufficient pAb or HRP to ensure the strongest RCI. Secondly, the optimum application amount of synthesized HAC nanoflowers was verified (Figure 3b). The strongest RCI occurred when the 10-fold dilution of synthesized HAC nanoflowers was applied to the detection, and the higher dilutions could have reduced the amount of HRP involved in the reaction. Thirdly, the amount of SMB determining the amount of mAb required for the capture of target *S. enteritidis* was also investigated (Figure 3c). Different amounts of SMB were tested, ranging from 10 μg to 30 μg, and 20 μg of SMB could obtain the strongest RCI. A higher amount of SMB may disrupt signal measurement. Fourthly, the effect of the pH value on HAC binding to the target was also studied (Figure 3d). The strongest RCI was acquired when the pH value was 7.0. The optimum detection conditions were concluded as follows: (1) pAb and HRP (total amount of 0.2 mg) with the mass ratio of 1:10; (2) 10-fold dilution of synthesized HAC nanoflowers; (3) 20 μg of SMB; (4) pH 7.0.

### 3.4. Detection of S. enteritidis Using HAC Nanoflowers

**Figure 4:**
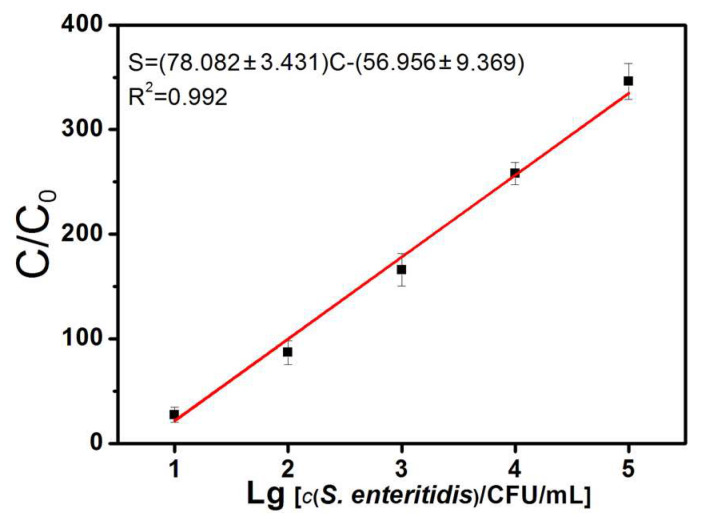
Linear calibration curve for the detection of *S. enteritidis*.

**Figure 5:**
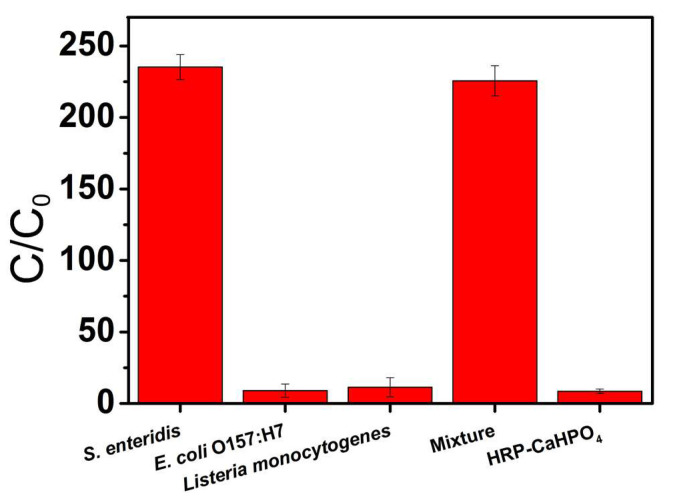
Specificity of the proposed method.

The detection of *S. enteritidis* was based on the combination of immunomagnetic separation and a sandwich-type immunoreaction. According to the process shown in Scheme 1, *S. enteritidis* was firstly captured by SMB-mAb and then the sandwich immunocomplex was formed by the introduction of HAC. After the magnetic separation, the HRP enriched on the HAC nanoflowers catalyzed the substrate, luminol, in the presence of H_2_O_2_ to produce the chemiluminescent signal. As shown in Figure 4, the RCI significantly increased as the concentration of *S. enteritidis* increased. The RCI was linearly dependent on the logarithm of the concentration of *S. enteritidis* ranging from 10 to 10^5^ CFU/mL with the LOD of 10 CFU/mL (3σ criterion) [43]. The linear equation was represented as S = (78.082 ± 3.431) C—(56.956 ± 9.369) (R^2^ = 0.992), where S is the RCI, C denotes the common logarithm of the concentration of *S. enteritidis*, and R^2^ is the correlation coefficient. In the previous study, only the morphology and size of such nanoflowers were characterized [40]. We have provided a more comprehensive analysis of their crystalline structure and composition, as well as the immobilization efficiency, content and proportion of HRP and Ab in the proposed HAC nanoflowers, which can contribute to the better understanding and application of this material. Moreover, the previous nanoflowers with a size of around ~5 μm were relatively large and could easily lead to non-specific adsorption. We modified the synthesis method to control the size of the proposed HAC nanoflowers to be around ~1.5 μm, and 1% BSA and 0.5% Tween-20 were added to the dilution buffer of the HAC nanoflowers to reduce the non-specific binding to the target, so as to ensure the precision of the proposed method. To assess the precision of the proposed method, a series of 10 consecutive measurements of *S. enteritidis* (10^3^ CFU/mL) were collected and a low relative standard deviation (RSD) of 4.2% was obtained, indicating the good precision of the proposed method. In addition, to obtain reproducible results, every step of the operation, from synthesis to detection, including the time, temperature, volume, concentration, feeding ratio, etc., should be exactly followed. The good reproducibility of the proposed method was also proven by the low RSD of 5.8% for six measurements of *S. enteritidis* (10^3^ CFU/mL) using the HAC synthesized at six different times. In terms of the procedure for the synthesis, compared with most of the nanomaterial-based biosensors in Table 1, such as graphene oxide, gold, magnetic, carbon, copper nanoparticles and polyvalent directed peptide polymers, this proposed synthesis method involves no organic or harmful reagents, has a less complicated conjugation and purification process and is only performed in one step, with green and mild coprecipitation. Regarding the aspect of the detection platform and time, the lateral flow strip provides a low cost and more rapid processing, but the sensitivity is not optimal. Although the smartphone-based biosensor achieves more flexibility and portability, as well as higher sensitivity and a wider linear range, it takes approximately two more hours to complete the detection, and there is no specific information about the precision and reproducibility of the method. On the basis of the sensitivity and linear range, the proposed method is more sensitive or has a wider linear range than most of the reported methods in Table 1. To evaluate the specificity of the proposed method, two non-target bacteria of *E. coli* O157:H7 and *Listeria monocytogenes* and a mixture of *S. enteritidis* and two non-target bacteria were detected (Figure 5). The RCI for the detection of *E. coli* O157:H7 and *Listeria monocytogenes* in the absence of *S. enteritidis* was similar to that of the blank. In contrast, in the presence of *S. enteritidis*, a relatively strong RCI was observed, suggesting the good specificity of the proposed method. Moreover, recovery experiments were conducted to further investigated the applicability of this method. Milk was spiked with *S. enteritidis* at the concentrations of 1 × 10^2^, 1 × 10^3^ and 1 × 10^4^ CFU/mL. The average recovery of spiked *S. enteritidis* was between 92.6% and 102.5% (Table 2), indicating the satisfactory application potential of the proposed method in milk.

## 4. Conclusions

In summary, this study reported a green, mild, simple and efficient method for the conjugation of HRP and Ab with CaHPO_4_ to prepare HAC bifunctional hybrid nanoflowers. The combination of a magnetic chemiluminescence immunoassay and HAC nanoflowers showed excellent sensitivity, specificity, accuracy and applicability for the detection of *S. enteritidis*. The proposed method provides a universal approach for the sensitive detection of foodborne pathogenic bacteria in milk.

## Figures and Tables

**Table 1:** Comparison of different methods for *S*. *enteritidis* detection.

Materials	Technique	Linear Range	LOD	Detection Time (h)	Reference
		CFU/mL	CFU/mL		
Copper nanoparticles	Fluorescent biosensor	50–10^4^	25	~2.6	[13]
Carbon nanoparticles	Lateral flow	10^2^–10^8^	10^2^	~0.25	[14]
Magnetic nanocomposite	Smartphone-based colorimetric immunosensor	1–10^6^	1	~3.4	[40]
Aptamer	Electrochemical biosensor	2.4–2.4 × 10^3^	3	~1.5	[44]
Gold	Surface plasmon resonance	0–10^7^	25	~0.61	[45]
Gold nanorods	Electrochemical immunosensor	1–10^5^	105	Not given	[46]
Magnetic nanoparticles	Lateral flow	10^3^–10^7^	10^2^–10^3^	~0.5	[47]
Polyvalent directed peptide polymer	Fluorescence microscope	10^2^–10^8^	10^2^	~2.0	[48]
HAC	Magnetic chemiluminescence immunoassay	10–10^5^	10	~1.5	This work

**Table 2:** Recovery of *S*. *enteritidis*-spiked milk samples.

*S. enteritidis* Added (CFU/mL)	*S. enteritidis* Found (CFU/mL)	Recovery (%)
1 × 10^2^	(0.926 ± 0.036) × 10^2^	92.6
1 × 10^3^	(0.945 ± 0.057) × 10^3^	94.5
1 × 10^4^	(1.025 ± 0.063) × 10^4^	102.5
0	Not found	-

## Data Availability

The data presented in this study are available on request from the corresponding author.

## Supplementary Materials

The following supporting information can be downloaded at: https://www.mdpi.com/article/10.3390/s23052779/s1, Figure S1: EDX spectrum of the HAC hybrid nanoflowers.
