# Supplementary material for: One-Pot Synthesis of Enzyme and Antibody/CaHPO4 Nanoflowers for Magnetic Chemiluminescence Immunoassay of Salmonella enteritidis
Source: Sensors (Basel). 2023 Mar 3;23(5):2779. doi: 10.3390/s23052779 (PMC10006971; doi:10.3390/s23052779)
Supplement: Supplementary file 1 [file sensors-23-02779-s001.zip › sensors-2227768-supplementary.pdf]

One-Pot Synthesis of Enzyme and Antibody/$CaHPO_4$ Nanoflowers for Magnetic Chemiluminescence Immunoassay of *Salmonella enteritidis*

Xingchu Mao and Ranfeng Ye *

College of Science, Huazhong Agricultural University, Wuhan 430070, China

* Corresponding author E-mail addresses:

ranfeng.ye@mail.hzau.edu.cn

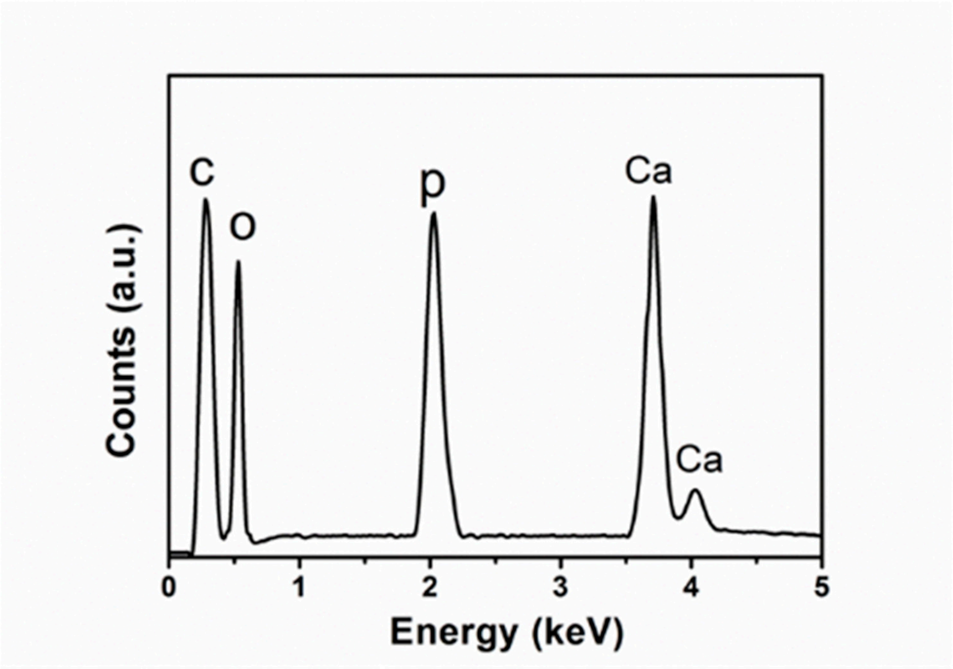

Figure S1. EDX spectrum of the HAC hybrid nanoflowers.
